# Modeling the adoption of medical wearable devices among the senior adults: Using hybrid SEM-neural network approach

**DOI:** 10.3389/fpubh.2022.1016065

**Published:** 2022-10-28

**Authors:** Zou Xinyan, Abdullah Al Mamun, Mohd Helmi Ali, Long Siyu, Qing Yang, Naeem Hayat

**Affiliations:** ^1^UCSI Graduate Business School, UCSI University, Kuala Lumpur, Malaysia; ^2^UKM - Graduate School of Business, Universiti Kebangsaan Malaysia, Bangi, Selangor, Malaysia; ^3^Global Entrepreneurship Research and Innovation Centre, Universiti Malaysia Kelantan, Kota Bharu, Kelantan, Malaysia

**Keywords:** wearable healthcare device, intention and adoption, unified theory of acceptance and use of technology, senior adult, public health

## Abstract

The world is witnessing an increasing number of senior adult residents who experience health issues. Healthcare innovation facilitates monitoring the health conditions of senior adults and reducing the burden on healthcare institutions. The study explored the effect of health improvement expectancy, effort expectancy, price value, perceived vulnerability, health consciousness, and perceived reliability on the intention and adoption of medical wearable devices (MWD) among senior adults in China. Furthermore, a cross-sectional design was adopted, while quantitative data was collected from 304 senior adults through an online survey. A hybrid approach of partial least square structural equational modeling and artificial neural network-based analysis technique was adopted. The findings demonstrated that health improvement expectancy, perceived vulnerability, price value, and perceived reliability significantly affected the intention to adopt MWDs. Moreover, the intention to adopt MWDs significantly positively affected the actual adoption of MWDs among senior adults. Although the moderating effect of the pre-existing conditions and income between the intention to use MWDs and actual adoption of MWDs was positive, it was not statistically significant. The artificial neural network analysis has proven that perceived reliability, price value, and vulnerability are the most critical factors contributing to the intention to use MWDs. The current study offered valuable insights into the factors affecting the intention and adoption of MWDs among senior adults. Following that, theoretical and practical contributions were documented to improve the ease of use and price value for the prospective users of MWDs. The correct healthcare policies could curtail the influx of senior adults into the hospital and empower these adults to track and manage their health issues at home.

## Introduction

Wearable devices (WDs) fall under the next phase of mobile accessories, which offer the utility to monitor fitness and medical healthcare devices (1). Fitness wearable devices monitor daily health fitness, which involves footsteps, distance covered, sleep duration, and daily diet intake (2). Young health-conscious users are attracted to fitness wearable devices, such as Fitbit, Jawbone, and Kids Guardian (3). On the other hand, medical wearable devices (MWD) assist in monitoring elders, diabetic, or cancer patients (4). Apple, Google, and Samsung have improved medical wearable devices to monitor blood glucose, genetic testing, and daily health monitoring, enabling personal healthcare and facilitating doctor-patient communication (5).

The MWD is a new technology that could provide an efficient and extensible method of responding to the growing needs for the care of senior adults and their independence (6). These MWDs are highly useful in monitoring the healthcare of senior adults, which is performed by measuring body temperature, heart rate, and blood pressure (4). It could also monitor the risk indicators for disease progression, reduction, and other life-threatening situations (7). Besides, monitoring the health of senior adults could reduce hospitalization, improve spiritual life, provide a healthier lifestyle, help prevent and manage emergency situations, and reduce mortality chances (8). The demand for MWDs among senior adults is expected to continue to expand. Two main types of MWDs are available for customers in the current market, wearable fitness and medical devices (9). Specifically, wearable fitness devices are more suitable for young adults, middle-aged adults, and healthy users (10), while wearable medical devices are more suitable for senior adults and unhealthy users (3). The most common MWDs in the China market are smart bands (e.g., FitBit Flex, Xiaomi, Huawei, Dido), smartwatches with health monitoring features in China including Xiaomi Watch, Apple Watch, Samsung Galaxy Watch, and Honor Watch, wearable blood pressure monitor/watch, glucose monitor and smart neck ring (e.g., Fineck) (9).

The MWD is valuable in people's lives, especially senior adults. Therefore, exploring the intention and actual adoption of MWDs among senior adults is essential (3). The market growth for the MWDs depends on the technology acceptance of the users and the importance of the users' health conditions. Despite the projected growth of MWDs, the users' acceptance of these MWDs has been slow (11). The literature work demonstrated that the continuous usage of MWDs declined to 70% after 6 months of the first purchase of an MWD, followed by a further decrease of 55% after 1-year usage (12, 13). The MWDs developers are currently working to achieve long-term engagement of the consumers, given that the penetration of the MWDs in the market has been reduced and the first generation of consumers has abandoned the usage of these MWDs (14). Hence, to enhance the understanding of the behavioral intention and actual adoption of consumers of the MWDs, an examination of the effect of relevant factors on the intention and actual adoption MWDs is essential (11).

A sharp increase in the adult population takes place in China. By the end of 2030, 25% of the total population would have consisted of people above 60 years old (15). The healthcare industry is multiplying in China. Besides, senior adults require necessary healthcare services at their doorstep and are willing to devote money, time, and energy to their health (9). These MWDs are gaining more popularity in China regarding monitoring and managing personal health or daily life using MWDs or other health check devices (15). The adoption of MWDs among senior adults reduces the healthcare industry's burden and improves senior adults' independence.

In the recent spread of the COVID-19 pandemic, senior adults are among the individuals who receive the most impact from the imposition of lockdown instigating movement control. Despite senior adults' need for medical treatment, hospitals are not offering services to general patients unless when the condition is critical. Thus, the option left is for the senior adults to take precautionary measures and use the MWD to track their health conditions. While senior adults are more prone to COVID-19 infection, using MWDs promotes preventive behavior and a sense of empowerment. Most senior adults face health conditions and need to monitor their health condition daily or several times a day. Given the potential benefits of MWDs, it is necessary to explore the intention and adoption of MWDs during the COVID-19 time period. Therefore, exploring the adoption of MWDs among the senior adults with the MWDs technological factors and personal health concerns among the perspective users became obvious. The current study aims to explore the intention and adoption of MWDs, including the factors of MWDs technology (e.g., health improvement expectancy, effort expectancy, price value, reliability) and personal health concerns (vulnerability and health consciousness) among the rising population of senior adults. However, the pre-existing health conditions and average income suggested that the relationship between intention to adopt and actual adoption is moderated.

## Literature review

### Theoretical foundation

Venkatesh developed the unified theory of acceptance and use of technology (UTAUT). Venkatesh et al. (16) included four constructs: performance expectancy, effort expectancy, social influence, and facilitating conditions. The original UTAUT model was extended with three additional constructs: price value, hedonic motivation, and habit. The model is termed as UTAUT2 model (17). Compared to other technology acceptance models, the UTAUT2 model is considered the most comprehensive model to examine the intention and adoption of new technology among consumers (5). Besides, the UTAUT2 model could be used to determine the complex and innovative technology acceptance and users' adoption pattern (17). Many studies of wearable technology have proven that compared to other technology acceptance models, the UTAUT2 model shows better predictive power (18). Therefore, the current study integrated three constructs from the UTAUT2 model, including performance expectancy, effort expectancy, and price value, to predict the adoption of MWDs among senior adults.

Health behavior could be explained by two main theories: the protection motivation theory (PMT) and the health belief model (19). The literature suggested that PMT was widely utilized in adopting mobile health (mHealth) and MWDs. Based on the user's adoption, PMT falls under two categories: (1) coping appraisal, which consists of self-efficacy, response efficacy, and response cost, and (2) threat appraisal, which consists of perceived severity and vulnerability (5). Furthermore, threat appraisal is based on perceived severity and perceived vulnerability. To be specific, perceived vulnerability refers to the possibility that the individual perceives that he could suffer from health threats high perceived vulnerability). It also mainly evaluates the situation's seriousness (20). For this reason, perceived vulnerability and health consciousness are significant predictors of the intention to use the healthcare systems.

Technology adoption is highly associated with the perception of product or service reliability (12). The MWDs are innovative healthcare products, while the consumers' confidence and perception of product quality harness the intention and accelerated acceptance of the MWDs (21). Personal factors play a significant role in developing health-related technology products and services (22). Notably, senior adults possess pre-existing health conditions, leading to adoption of self-care health systems (23). Although the technology is priced above the existing products available in the market (20), personal income or available resources facilitate the prospective users to accept and use innovative healthcare technologies, such as MWDs (24, 25).

### Hypotheses development

#### Health improvement expectancy

Typically, people expect effective disease prevention and health management through healthcare applications. In this case, HIE could be defined as the individuals' perception of improving health conditions and quality through MWDs (23). The individuals who perceive that behavior is effective often tend to repeat the behavior and achieve the desired goal (26). Hence, individuals achieve their goals of HIE through the applications of MWD, such as disease prevention and management of health. Nonetheless, individuals are inclined to encourage others to apply these technologies if the results exceed their expectations (8). Moreover, Cimperman et al. (23) found that the intention and adoption of long-distance medical services for senior adults and HIE directly impacted the intention and adoption of long-distance medical services. The HIE could inspire the behavioral intention to adopt MWD (7). Based on the literature, HIE could predict the intention and adoption of MWD among senior adults (3). The following hypothesis was proposed:

H1: HIE impacts the intention to use MWD.

#### Effort expectancy

Effort expectancy refers to the ease of use of technology (16). In the MWDs context, EEX reflects the perception of individuals toward the convenience of using MWDs (1). Nevertheless, the ease of use of the technology significantly influences consumers' intention and adoption behavior, particularly in the initial stage of technology commercialization (23). For senior adults, MWDs remain a relatively new technology. Therefore, it is critical to determine whether the senior adults perceive that the MWDs are easy to learn and use, including the possibility for the MWDs to influence the intention and decisions for adoption (4). Hence, the EEX of MWD is expected to positively impact the behavioral intention for adopting MWDs among senior adults (5). In addition, given that MWDs are new to users, ease of use significantly affects behavioral intention. Dwivedi et al. (22) demonstrated that effort reduction is a significant factor in adopting novel technologies. Sun et al. (27) highlighted that technology level EEX directly affects the behavioral intention to adopt a system of mobile health monitoring, mobile health, and service of e-health. The following hypothesis was proposed:

H2: EEX impacts the intention to use MWD.

#### Price value

Price value refers to the difference perceived by the customer between the monetary cost of the technology and the perceived benefits derived from the use of technologies (17). The PRV represents the acceptable degree of price and the best value for the products (28). Compared to traditional healthcare services, mHealth or MWDs could offer the most cost-effective healthcare services by reducing in-person visits and hospitalisations (19). In this situation, individuals who use this technology tend to switch to competitive services (24). The literature suggests that PRV positively affects the intention and adoption of wearable technology (22). Nevertheless, given the significance of believing that the perception of associated cost influences technology adoption, it is crucial to explore the impacts of PRV on the intention and adoption of mHealth-based applications (24). Therefore, PRV could be one of the essential factors affecting the behavioral intention to adopt MWDs among senior adults (22, 29). Based on the empirical evidence, the following hypothesis was suggested:

H3: PRV impacts the intention to use MWD.

#### Perceived vulnerability

Perceived vulnerability is defined as the possibility for the individual to feel that he might suffer from health-related issues or health threats (5). In this situation, adopting related healthcare technology is expected to prevent or manage health threats (19). However, if the individual regards these threats as severe, they would be inclined to adopt health information technology to prevent or avoid a health threat (20). Gao et al. (5) examined the adoption of mHealth based on the PMT, which includes threat appraisal and coping appraisal. Furthermore, it was found that the factors of threat appraisal affected the behavioral intention to adopt mobile health. Besides, PVU is one of the factors depicting the threat appraisal. Sun et al. (27) and Gao et al. (5) highlighted that PVU significantly impacts healthcare technology intention and adoption. Accordingly, past research works have proved that PVU positively impacts the intention and adoption of the technology of healthcare (27, 30). Hence, the following hypothesis was proposed:

H4: PVU impacts the intention to use MWD.

#### Health consciousness

Health consciousness denotes the individuals' concern for personal health (25). Healthcare marketing has advocated a psychology-oriented approach to preventing behaviors in healthcare. Consumers who adopt health-oriented lifestyles and are health-conscious are more likely to use MWDs to monitor health indicators than consumers who are not (25, 29). Additionally, senior adults who care about their health are interested in monitoring their health information and learning how to use the new health technology to manage their health (7). Cho et al. (25) recorded that HCO significantly affected the behavioral intention to adopt mHealth. To illustrate, individuals with health consciousness show more engagement in obtaining the correct information for personal health monitoring. Sergueeva et al. (11) indicated that HCO is one of the critical factors in predicting health-related precautionary behaviors (11). Based on this literature, the following hypothesis was formulated:

H5: HCO impacts the intention to use MWD.

#### Perceived reliability

Reliability refers to the extent to which a customer believes that the new technology could show an accurate and consistent performance of a task (31). The PRE is a noteworthy predictor of customer satisfaction and technology intention to use (29). Furthermore, it was established with the technical ability to deliver the promised services safely, accurately, and steadily (32). Reliability is the most critical factor in selecting healthcare services, given that improper treatment might lead to life-threatening situations (33). In addition, the consumers would find the technology reliable and trustworthy, with the highest possibility of being adopted if the system is user-friendly (29). Warrington et al. (34) postulated that the technology PRE influences intention and technology adoption. Additionally, Gao et al. (5) highlighted that those wearable healthcare devices perceived that quality significantly impacts the intention and adoption of MWDs. Wang et al. (1) documented that PRE positively impacts the acceptance of healthcare wearable devices and allied ICT-based services. Based on this discussion, PRE could positively affect the intention to adopt MWDs among senior adults.

H6: PRE influences the intention to use MWD.

#### Intention and adoption of WPD

Behavioral intention refers to the extent of an individual's perceived willingness to use new technology (35). It is a vital predictor of the actual adoption of health-related technology. Alam et al. (29) argued that intention is the best predictor for adopting health-based wearable devices, mobile devices, or other domains. Senior adults require urgent health attention at a personal level, so the intention to use health-based personal devices predicts the adoption of MWD (12). A consumer is more likely to accept new technology when the behavioral intention is high. Accordingly, the following hypothesis was made:

H7: The intention to use MWD influences MWDs adoption.

### Moderating effect of pre-existing conditions and average monthly income

In the past literature, the relationship between independent and dependent variables or mediators (behavioral intention) is inconsistent (21). The differences in these findings could be attributed to past research, which overlooked the moderating effects of several critical factors, including age, gender, experience, and income for adopting health technology (6, 20). Several studies demonstrated that income level affected the actual adoption of wearable devices and information systems (10). Pre-existing conditions are becoming more common among the populations of traumatized senior adults, which could be a critical factor in the increasing mortality (19). The common pre-existing conditions of senior adults with high blood pressure, diabetes, cardiovascular disease, cerebrovascular disease, and myocardial infarction could influence the intention of using wearable healthcare devices (4). Hence, seniors with pre-existing conditions who wish to prevent or avoid health threats are more likely to adopt healthcare technology services to monitor their health indicators, such as mHealth and wearable devices (7). Based on the discussion above, income level and pre-existing conditions were proposed as the moderators to examine the moderating impact of behavioral intention to adopt the MWDs of senior adults.

**Figure 1:**
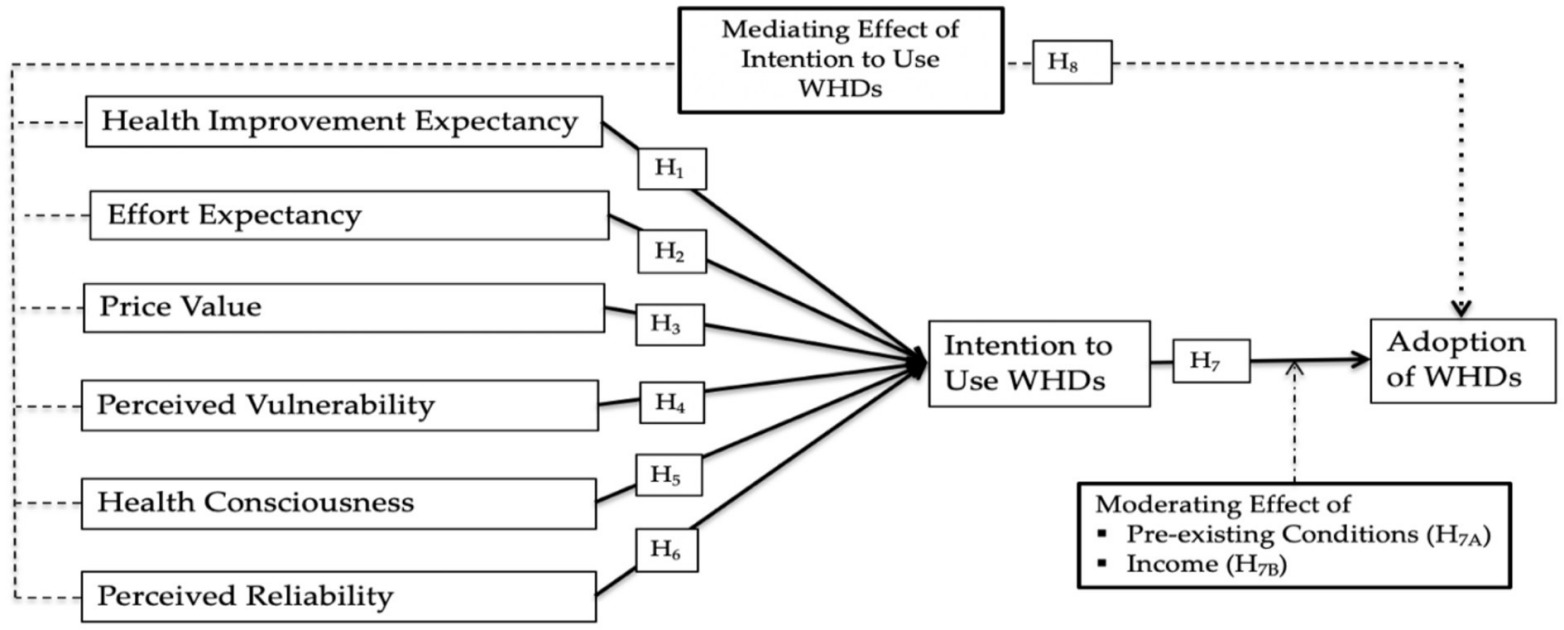
Research framework.

All associations hypothesized are accessible in Figure 1.

## Research methodology

### Data collection and sample selection

Convenience sampling is non-probability sampling used to collect data from individuals in proximity. This sampling is conveniently available and allows the selection of respondents within the researcher's access (36). The WJX online survey form was used for data collection. The questionnaire collected responses from senior adults (> 50 years old) in China who have gained experience in using wearable devices to monitor their health. As a result, 304 questionnaires were collected for the study.

### Measurement and scales

The measurement scale for the study was developed from established and respected scales. The questionnaire items were utilized to measure the variables and sources shown in Supplementary material-Appendix 1. The questionnaire was designed in English and translated to Chinese to allow the respondents to understand better and effectively respond to the questionnaire items (36). A five-point Likert scale was utilized for all the questionnaire items related to the exogenous variables, while a seven-point Likert scale was employed for the endogenous variables. Using different Likert scales for input and outcome variables assisted in addressing the issue of common method variance in the research design stage (37).

### Common method variance

Harman's one-factor test was applied to determine the effect of CMV as a diagnostic technique (37). The single factor accounted for 36.538%, which was below the recommended threshold of 50% in Harman's one-factor test and led to the approval of the negligible influence of CMV in this study. Furthermore, the study evaluated the common method variance by following Kock's (38) recommendation to test the full collinearity of all the constructs. All the study constructs regressed on the common variable, the variance inflation factor (VIF) value for health improvement expectancy (2.924), effort expectancy (2.915), price value (3.007), perceived vulnerability (3.438), health consciousness (2.486), perceived reliability (3.345), intention to use WMDs (3.268), and adoption of MWDs (2.967). The value of 3.5 indicated the absence of bias from the single-source data.

### Multivariate normality

Multivariate normality for the study data was assessed with the Web Power online tool. The calculated Mardia's multivariate skewness and kurtosis coefficient and *p*-values demonstrated that the non-normality issue was present in the study data, given that the *p*-values were below 0.05 (39).

### Data analysis method

This study applied the PLS-SEM technique to verify the proposed model and examine the proposed hypotheses using SmartPLS 3.0. Many studies have validated and generally employed the PLS-SEM technique for hypothesis testing (40). The characteristic of this technique is present in the flexibility of data allocation, which is suitable for small-size sampling (38). Inspecting the construct reliability, convergent validity, and discriminant validity are critical before examining the structural model (41). Furthermore, Cronbach's Alpha measured the reliability, while Dillon-Goldstein's rho, composite reliability, and average variance extracted (AVE) measured the internal consistency reliability (40). Discriminant validity was examined by the Fornell-larcker criterion, heterotrait-monotrait ratio (HTMT), and loadings and cross-loading. The path coefficients were used to test the hypothesis (41), which also involved the use of beta (coefficients), confident interval, *t*-value, and *p*-value (40).

### Artificial neural network analysis

Artificial neural network (ANN) analysis is a quasi-analytical technique comprising three layers: input, output, and hidden (42). The input and output neurons are linked through the hidden layer, which functions the same way as the human brain block-box (43).

The ANN analysis is a non-compensatory analytical approach that uses a deep learning method with three layers: input, output, and hidden (4). The underlying layer connects input neurons to output neurons. Notably, the buried layer has the same function as the human brain block-box (44). The information is separated into three categories: training, testing, and holding out the sample.

The predictive score is calculated by summing the training and testing data's root mean square errors (RMSE) (44). The prediction accuracy would increase with higher significance in the difference of the RSME scores between training and testing data (42). Sensitivity analysis was performed to evaluate the relative influence of each external factor. Following that, the influence of each exogenous on the endogenous structure is demonstrated through the normalized relevance of each exogenous (4). The average synaptic weights assist in the understanding of the contribution of input and hidden layers to output (43).

## Findings

### Demographic characteristics

As shown in Table 1, the majority of the 304 respondents in this study were male (53.3%). Over half of the respondents (55.6%) were aged between 50 and 59 years old, while 44.1% of the respondents were above 60 years old. Most of the respondents originated from Shanghai (15.8%), Shandong (15.1%), Guangxi (14.5%), Hunan (12.5%), Guangdong (12.5%), and Beijing (11.8%).

**Table 1:** Demographic characteristics.

	***N***	**%**		***N***	**%**
***Gender***			***Education***		
Male	162	53.3	Secondary school certificate	39	12.8
Female	142	46.7	Diploma	58	19.1
Total	304	100	Bachelor degree or equivalent	146	48.0
			Master's degree	30	9.9
***Age***			Doctoral degree	31	10.2
30–39	1	0.03	Total	304	100
40–49	0	0.00			
50–59	169	55.6	***Average monthly income***		
60 Above	134	44.1	Below CNY 2,500	23	7.6
Total	304	100	CNY 2,501-CNY 5,000	84	27.6
			CNY 5,001-CNY 7,500	83	27.3
***Living province***			CNY 7,501-CNY 10,000	38	12.5
Beijing	36	11.8	CNY 10,001-CNY 12,500	45	14.8
Shanghai	48	15.8	More than CNY 12,500	31	10.2
Guangdong	38	12.5	Total	304	100
Guangxi	44	14.5			
Zhejiang	18	5.9	***Do you have any pre-existing conditions?***		
Shandong	46	15.1	Had types of medical diseases	73	24
Hunan	38	12.5	I had chronic conditions	94	30.9
Jiangsu	17	5.6	I had high blood pressure	46	15.1
Others	19	6.3	I had cardiovascular diseases	65	21.4
Total	304	100	Others	26	8.6
			Total	304	100

Most of the study respondents achieved Bachelor level education (48%), 19.1% received diploma level education, 12.8% obtained secondary school level education, and 10.2% of respondents obtained doctoral level of education. Following that, 27.6% of the respondents gained an average monthly income of CNY 2,501–5,000, 27.3% of the respondents had an average monthly income of CNY 5,001–7,500, 14.8% of the respondents had an average monthly income of CNY 10,001–12,500, 12.5% of the respondents received an average monthly income of CNY 7,501–10,000, 10.2% of the respondents received the monthly income of higher than CNY 12,500. Among the respondents, 30.9% were faced with chronic conditions, 24% had several medical diseases, 21.4% were diagnosed with cardiovascular disease, and 15.1% were reported with blood pressure issues.

### Reliability and validity

In PLS-SEM, Cronbach's alpha (CA) usually is the criterion for internal consistency reliability. However, besides the CA, composite reliability (CR) is also used as the criterion to measure internal consistency reliability. Hence, the reliability of the construct was determined by CA and CR. With higher values of CR between 0 and 1, the reliability level would increase (41). Based on the results of the measurement model in Table 2, the CR values of the variables of the structural model were higher than 0.70, which confirmed the construct reliability. The generally acceptable values of CA range from 0.60 to 0.70, while values higher than 0.70 are considered a good level of reliability (40). As a result, the CA values of the variables of the structural model were over 0.688, leading to acceptable CA values of the variables of the structural model. Besides, the average variance extracted (AVE) was used to determine the construct convergent validity. Simultaneously, the AVE scores should exceed 0.50 to confirm convergent validity (40). As a result, all the constructs exhibited an AVE higher than 0.50, confirming convergent constructs' validity.

**Table 2:** Reliability and validity.

**Variables**	**No. of Items**	**Mean**	**SD**	**CA**	***Rho_A***	**CR**	**AVE**	**VIF**
HIE	4	4.228	0.588	0.695	0.695	0.814	0.522	2.820
EEX	4	4.246	0.598	0.704	0.706	0.818	0.530	2.848
PRV	4	4.265	0.617	0.718	0.720	0.826	0.542	2.878
PVL	5	4.253	0.592	0.698	0.698	0.815	0.524	3.400
HCO	4	4.192	0.568	0.674	0.677	0.803	0.505	2.475
PRL	5	4.305	0.591	0.688	0.691	0.810	0.533	3.029
IMWD	5	5.819	0.887	0.721	0.722	0.827	0.544	1.103
AMWD	4	5.778	0.882	0.689	0.690	0.811	0.518	

HIE, Health Improvement Expectancy; EEX, Effort Expectancy; PRV, Price Value; PVU, Perceived Vulnerability; HCO, Health Consciousness; PRL, Perceived Reliability; IMWD, Intention to Use MWD; AMWD, Usage of MWD; SD, Standard Deviation; CA, Cronbach's Alpha; DG rho, Dijkstra and Henseler's rho; CR, Composite Reliability; AVE, Average Variance Extracted; VIF, Variance Inflation Factors.

**Source:** Author's data analysis.

For the evaluation of the variables' variance inflation factor (VIF), it was suggested in past research that the values of VIF should be lower than 3.3 (41). Given that the values of VIF of all variables were lower than 3.3, no multicollinearity issues were present (38). The values of CA, CR, AVE, and VIF are shown in Table 2.

The discriminant validity was examined using Cross-loading and Fornell-Larcker criteria. The first method of evaluating the discriminant validity of the constructs is cross-loading, which recommends that the outer loading of the constructs should be higher than the cross-loadings. This is followed by the Fornell-Larcker criterion, which is the second way to examine the construct discriminant validity (refer to Supplementary material-Appendix 2) by comparing the square root of AVE values with the correlations among the variables of constructs (40). The discriminant validity was evaluated by cross-loading, which exhibited a strong connection among the constructs with its items, as shown in Supplementary material-Appendix 2. According to the results of the Fornell-Larcker criterion shown in Appendix 2, it was proven that the validity of the discriminant of the construct as a result of the construct loadings was higher compared to other constructs (refer to Supplementary material-Appendix 2).

### Hypothesis testing

The path coefficients represent the relationships of the structural model. Table 3 presents the results of the proposed model variables, which represent the relationship between six independent variables and the intention to adopt MWD, including the relationship between the intention to adopt MWD and the actual adoption of MWDs. Besides the *p* < 0.05, the hypothesis was accepted. However, the hypothesis was rejected upon the *p* > 0.05 (40). Based on the results in **Table 5**, the path coefficients of HIE on the intention to adopt MWD achieved the acceptable *p*-value, which offered acceptance support for H1. Moreover, the path coefficients of EEX on the IMWD were insignificant and rendered no support to accept H2. Following that, the path between the PRV on the IMWD achieves a significant level and suggests the acceptance of H3. Subsequently, the path between PVL on the IMWD suggested the achievement of statistical support to accept the H4. Similarly, the path coefficient for HCO on the IMWD also supported the acceptance of H5. The path between PRL on the IMWD achieved the statistical support to accept H6. Apart from that, the path between the IMWD on the AMWD offered significant statistical support to accept the H7. Overall, the path analysis results are illustrated in Table 3.

**Table 3:** Path coefficients.

**No**.	**Path**	**Coefficients**	**CI-Min**	**CI–Max**	***t***	***p***	***r*** **^2^**	***f*** **^2^**	**Decision**
H_1_	HIE → IMWD	0.134	0.035	0.237	2.205	0.014		0.018	Accept
H_2_	EEX → IMWD	0.031	−0.057	0.120	0.577	0.282		0.001	Reject
H_3_	PRV → IMWD	0.200	0.105	0.289	3.577	0.000		0.040	Accept
H_4_	PVL → IMWD	0.183	0.084	0.280	3.050	0.001		0.033	Accept
H_5_	HCO → IMWD	0.111	0.029	0.192	2.234	0.013		0.014	Accept
H_6_	PRE → IMWD	0.270	0.178	0.356	5.017	0.000	0.642	0.083	Accept
Factors affecting the adoption of MWDs									
H_7_	IMWD → AMWD	0.724	0.529	0.807	8.135	0.000	0.488	0.898	Accept

HIE, Health Improvement Expectancy; EEX, Effort Expectancy; PRV, Price Value; PVU, Perceived Vulnerability; HCO, Health Consciousness; PRE, Perceived Reliability; IMWD, Intention to Use MWD; AMWD, Usage of MWD.

**Source:** Author's data analysis.

### Moderating effects

**Figure 2:**
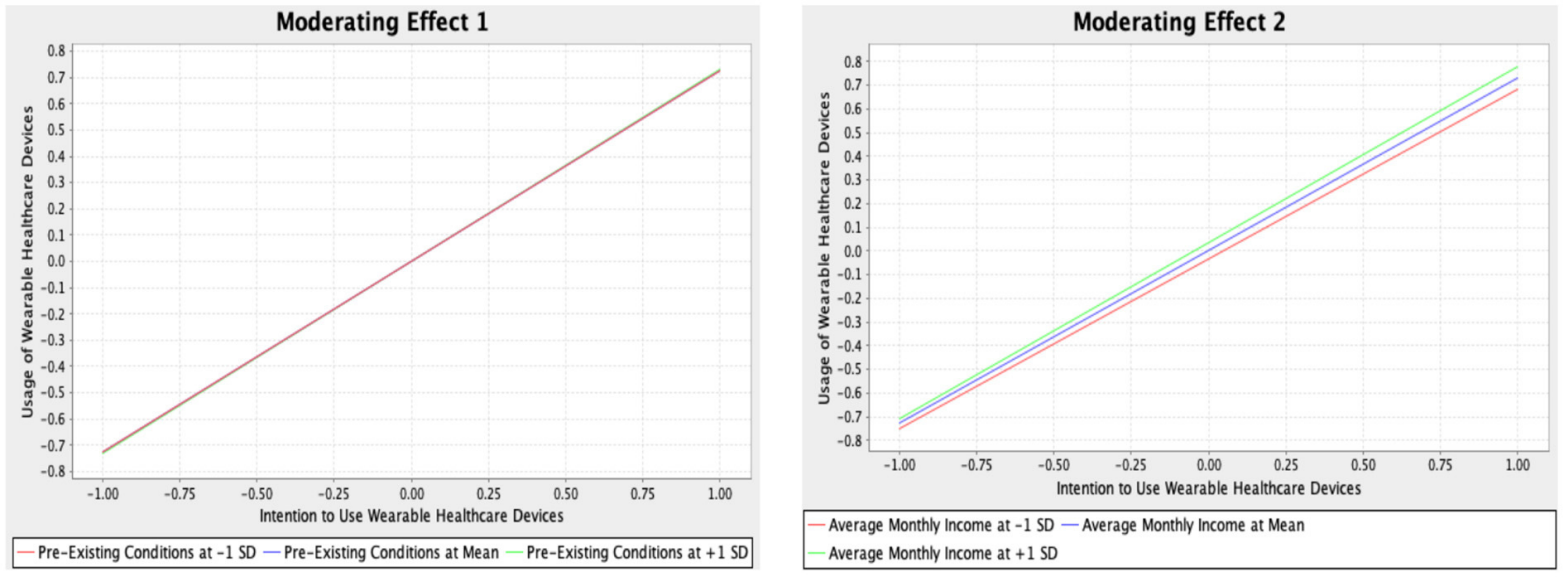
Moderating effects.

The moderator could directly impact the relationship between the input and dependent variables. In this study, pre-existing conditions (PRC) and average monthly income (AMI) moderators moderated the relationship between the intention to use MWDs and the actual adoption of MWDs. It was indicated from the result that the PRC made insignificant moderation on the association between the IMWD and MWDs. Subsequently, the AMI insignificantly moderated the relationship between the IMWD and AMWD. The analysis suggested that the PRC and AMI did not moderate the impact of IMWD on AMWD. The moderation analysis is presented in Table 4 and shown in Figure 2.

**Table 4:** Moderating effects.

**Path**	**Coefficients**	**CI-Min**	**CI-Max**	***t***	***P***	**Decision**
PRCxIMWD → AMWD	0.080	−0.073	0.194	0.984	0.163	No moderation
AMIxIMWD → AMWD	−0.046	−0.191	0.093	0.529	0.299	No moderation

PEC, Pre-Existing Conditions; AMI, Average Monthly Income.

**Source:** Author's data analysis.

### Neural network analysis

This research employed the multi-layer perception (MLP) ANN, consisting of three layers: input, hidden, and output (42). The feed-forward-back propagation (FFBP) with MLP ANN was employed. Following that, a 10-fold ANN model in the SPSS neural network algorithm was selected (44). While 70% of the data was utilized for training, 30% of the data was used for testing. Average synaptic weights of the input and hidden neurons of the ANN are presented in Supplementary material-Appendix 3.

The prediction accuracy was evaluated with the RMSE score of the model (4). Based on Table 5, the results presented high predictive accuracy, given that the RMSE values of training and testing segments of data were close.

**Table 5:** RMSE values of artificial neural networks (*N* = 304).

	**Sample** **size** **(testing)**	**Sample** **size** **(testing)**	**Rmse** **(training)**	**Rmse** **(testing)**	**Sse** **(testing)**	**Sample** **size** **(training)**	**Sample** **size** **(testing)**	**Rmse** **(training)**	**Rmse** **(testing)**	**Sse** **(testing)**
***Model A: Factors effecting IMWD***						***Model B: Factors effecting AMWD***				
1	223	81	0.390	0.161	15.143	219	85	0.367	0.218	12.771
2	218	86	0.290	0.286	11.906	215	89	0.259	0.617	11.235
3	211	93	0.252	0.332	15.155	205	99	0.306	0.330	15.656
4	201	103	0.280	0.258	12.850	219	85	0.254	0.596	12.763
5	217	87	0.260	0.361	10.071	202	102	0.311	0.304	20.408
6	211	93	0.336	0.179	12.359	208	96	0.425	0.195	19.152
7	209	95	0.285	0.235	14.269	212	92	0.267	0.497	13.577
8	212	92	0.249	0.335	14.657	199	105	0.292	0.350	14.430
9	219	85	0.282	0.402	11.203	215	89	0.332	0.258	12.564
10	209	95	0.296	0.407	12.069	207	97	0.312	0.317	15.661
		Mean	0.292	0.295	12.968		Mean	0.312	0.368	14.821
	Standard deviation		0.042	0.086	1.760	Standard deviation		0.052	0.150	2.971

**Source:** Author's data analysis.

**Figure 3:**
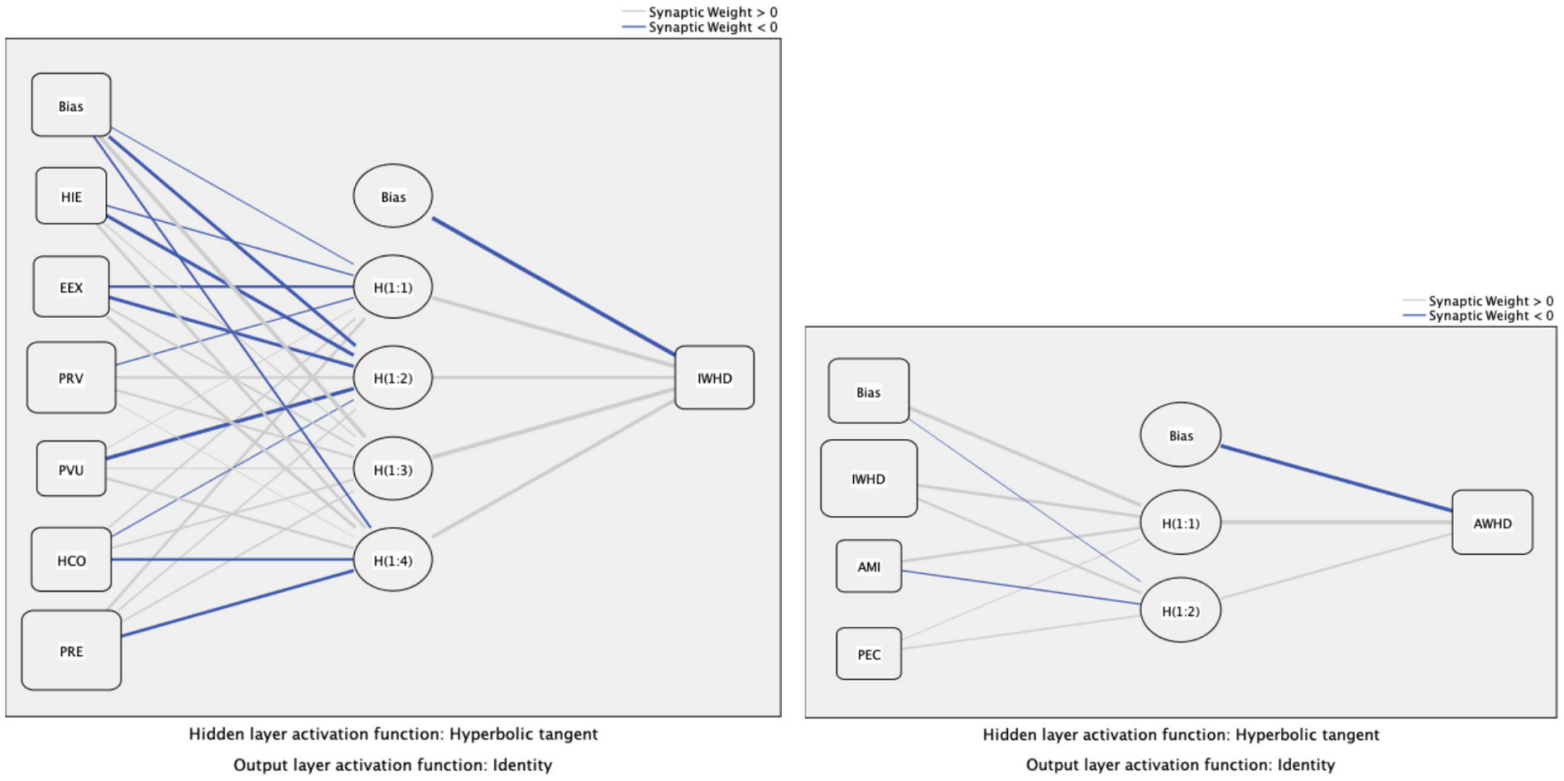
Hidden layers.

The relative values of RMSE for the training and testing of Model A and Model B demonstrated that the data achieved higher predictive accuracy (43). The ANN 1 model could predict the intention of using MWD by 97.7% through the goodness of fit. In the ANN model 2, the goodness of fit amounted to 97.5%, while the intention to use MWD showed the most significant contributing factor to the use of MWD. Following that, the sensitivity analysis was assumed to appraise the influence of each input variable in the model for wearable health devices (42). Normalized importance scores for every input construct were gained with the percentage fraction of the relative importance of each input neuron, which was divided by the highest relative importance (4). The result demonstrated that the five most significant contributing factors to the intention to use wearable health devices included perceived reliability, price value, perceived vulnerability, health consciousness, and effort expectancy. The evaluations are illustrated in Table 6. Models 1 and 2 with hidden layers are presented in Figure 3 below.

**Table 6:** Sensitivity analysis.

**Network**	**HIE**	**EEX**	**PRV**	**PVU**	**HCO**	**PRE**	**IMWD**	**AMI**	**PRE**
	***Factors effecting IMWD***						***Factors effecting AMWD***		
1	0.261	0.106	0.119	0.179	0.077	0.258	0.943	0.021	0.036
2	0.143	0.128	0.309	0.128	0.067	0.226	0.975	0.009	0.016
3	0.155	0.064	0.264	0.127	0.036	0.354	0.972	0.013	0.016
4	0.039	0.082	0.215	0.074	0.035	0.554	0.987	0.004	0.009
5	0.067	0.024	0.236	0.150	0.053	0.470	0.968	0.012	0.020
6	0.136	0.038	0.170	0.074	0.036	0.545	0.987	0.003	0.010
7	0.123	0.052	0.078	0.268	0.037	0.441	0.973	0.011	0.017
8	0.091	0.130	0.228	0.081	0.162	0.309	0.958	0.006	0.036
9	0.054	0.150	0.223	0.117	0.033	0.422	0.991	0.003	0.007
10	0.169	0.048	0.337	0.081	0.049	0.317	0.984	0.013	0.004
Mean importance	0.1238	0.0822	0.2179	0.1279	0.0585	0.3896	0.9672	0.0142	0.0187
Relative importance	31.77	21.09	55.92	32.82	15.01	100	100	0.95	1.75

HIE, Health Improvement Expectancy; EEX, Effort Expectancy; PRV, Price Value; PVU, Perceived Vulnerability; HCO, Health Consciousness; PRE, Perceived Reliability; IMWD, Intention to Use MWD; AMWD, Usage of MWD; PEC, Pre-Existing Conditions; AMI, Average Monthly Income.

**Source:** Author's data analysis.

## Discussion

The study analysis recorded that senior adults HIE had a significant impact on the intention to adopt MWD, with the result accepting H1. Furthermore, the study result coincided with the result reported by Lee and Lee (3), in which the health-related improvement expectancy of the individuals built the intention. The personal inclination to lead a healthy lifestyle and reduce health issues, including self-service technologies, empowers senior adults or patients to keep track of health indicators and promptly predict health problems. Following that, the study proposed to test the impact of the MWD effort expectancy on the intention to use MWD. However, it was proven in this study that the MWD effort expectany had an insignificant influence on the intention to use MWD, which offered substance for not supporting H2. Notably, this study was in line with the finding suggested by Talukder et al. (4), in which many users struggled to understand and use the MWDs. To be specific, most MWD users faced challenges in using MWD and taking the most advantage of its use. Even though the personal technical competence and technology experience facilitated the users (45), most senior adults were not highly experienced in the technologies, which was indicated through their struggle in using MWD. Rather than the ease of use, the effort expectany for MWD suggested the uneasiness or difficulty of using the MWD.

The study demonstrated that price value significantly affected the intention of using MWD among senior adults. Therefore, support for accepting H3 was made. The study result was in line with the result presented by Beh et al. (20) that the perception of value was derived from the use of technology and the price provided for it. As a technology, MWD offers more value compared to the prices paid, while the general consumers' perceived price value suggests the intention to use MWD. This study indicated that perceived vulnerability significantly influenced the intention to adopt MWD, which supported the acceptance of H4. Notably, this study was in line with Beh et al.'s (20) finding that the individuals' perception of health susceptibility formed the intention to use the health monitoring devices at personal convenience. Additionally, senior adults possessed higher perceived vulnerability and constantly sought technological devices which could facilitate health conditions daily at the convenience of home. Gao et al. (5) suggested that smart wearable healthcare devices are popular among seniors and young adults.

It was suggested from the result that health consciousness affected the intention to use the MWD, which advocated the acceptance of H5. Preeminent health concerns were observed from senior adults who constantly sought support to achieve constant health monitoring at the convenience of home or office. Personal health consciousness suggested using wearable devices to monitor daily physical activities and promote personal health conditions. Sergueeva et al. (11) presented empirical evidence that the health consciousness empowers senior adults' intention to adopt healthcare wearable technology devices to monitor and manage personal health issues. Routine health checking is essential for a healthy life and promotes well-being (29).

It was indicated from the results that perceived realiability is one of the crucial predictors of the intention to adopt MWD. The results were in line with the past results, in which perceived realiability had a significant effect on the intention to adopt healthcare technologies, such as mHealth services (12). As previously stated in the HCO findings, product reliability is critical before the consumer decides to buy MWD. Furthermore, given that perceived realiability reflects the technology's reliability, safety, and accuracy, it is crucial for the user's satisfaction and intention to adopt the technology (32). When consumers consider the adoption of a wearable device, product reliability, authenticity, and safety are the utmost concern. The perceived realiability is also the essential factor influencing consumers' intention to adopt health services (29).

The current study's findings verified the positive relationship between behavioral intention and actual adoption of MWDs among senior adults (H7). The findings were also consistent with past research, which identified a significant relationship between behavioral intention and actual adoption of wearable technologies, such as mHealth apps (29), the technology of wearable health monitoring (24), and wearable devices for patients (12). It was indicated from the past and current findings that a stronger intention to adopt MWDs contributed to a higher tendency to adopt these devices (12, 24).

The study's findings demonstrated that pre-existing conditions and income did not moderate the relationship between the intention to use MWDs and the actual adoption of MWDs. The findings regarding income in the study were not consistent with the past study, which recorded that income moderated the relationship between the intention to use healthcare technology and actual adoption of healthcare technology, such as the management system of mobile chronic diseases (10). Senior Chinese adults' average annual pension income was approximately CHY 38,000 (roughly RM 60,000) in 2018 (15). Similarly, the price of wearable devices in the market, such as the Xiaomi smartwatch (CHY 659) and Huawei (CHY 639), was also affordable (9).

Based on the demographic profile of the current study, most of the respondents resided in Beijing, Shanghai, and Guangdong, where the income is generally higher compared to other provinces in China. Besides, most respondents' monthly average income was higher than CHY 5,000. It was concluded that income did not moderate the relationship between the intention to use MWD and actual adoption of MWD among senior adults due to their capability to pay for the MWD price.

Nonetheless, past studies did not highlight the pre-existing conditions affecting the relationship between the intention to use MWD and the actual adoption of MWD. Based on the demographic profile, all 304 respondents suffered from certain levels of disease, such as high blood pressure and cardiovascular diseases. The respondents could choose to go to the hospital for regular check-ups, given that most senior adults in China have insurance, and the insurance company would pay for the expenses (10). Besides, the findings of the pre-existing conditions of this study were inconsistent with past studies, which identified that the risk of death from trauma could be increased by the pre-existing conditions (19). Provided that the senior adults with the disease did not necessarily choose to use MWD to monitor their health, the pre-existing conditions did not moderate the relationship between the intention to use MWD and the actual adoption of MWD.

The ANN analysis suggested that perceived reliability, price value, and vulnerability were three significant contributors that formed the intention of using the MWD. The ANN results were consistent with the PLS-SEM findings; however, the intention to use the MWD remained the most significant contributor to the adoption of MWD.

## Conclusion

The existing work proposes to determine the formation of intention and adoption for MWDs, with the perceived technological factor of health improvement expectancy, effort expectancy, price value, and reliability with the personal health concerns (like vulnerability and health consciousness) among the senior Chinese adults. Nevertheless, the association between the intention and adoption of MWDs is moderated by the health conditions and average income. The study consequences confirm that the health improvement expectancy, price value, reliability, vulnerability and health consciousness significantly influence the intention to adopt MWDs. The intention to adopt MWDs suggestively influence the adoption of MWDs.

### Theoretical implications

The current study contributed to the theory by combining the UTAUT and PMT to explore the formation of intention and adoption of MWD. It was also suggested that the reliability and price values were significant predictors of behavioral intention for senior adults. The moderating effect of personal income and pre-existing conditions did not influence the adoption of MWD. Apart from that, the integrated study model demonstrated the exploratory power of understanding the senior adults' intention to adopt MWDs.

Most previous studies focused on mHealth apps and wearable fitness devices (2, 29) or the younger generations and patients (12, 33). This study focused on the senior adult population and extensively explored the variables impacting the intention and adoption of MWD among senior adults. Through this action, the current study contributed to implementing innovation and purposes of healthcare behavior (4).

### Practical implications

Several important practical implications for the healthcare industry were present in this study. Specifically, the results suggested that MWD development should harness the quality and reduce prices to improve the price values of MWD. Furthermore, the social development agencies and MWD managers are required to increase the adoption of the MWD among senior adults based on a higher perception of MWD product reliability and value of money. Developers also need to improve the ease of use for MWD, given that many senior adults do not find it convenient to use the existing MWD. The correct instructional manual or video guideline must be prepared to educate the senior adults and reduce the efforts to understand and effectively use the MWD (19).

With the aging population, the interest in healthcare among senior adults has increased significantly, increasing the demand for intelligent healthcare services with self-management (4). Nowadays, various smart MWDs are available at different prices, such as SmartBand, Smartwatches, and Wearable Blood Pressure Monitor. It was indicated that senior adult users placed significant importance on the price value of wearable healthcare technologies and the best quality features. Hence, suppliers of MWDs or salespeople of MWDs should explain to the consumers the functionality of the products and the benefits of using the products to assure the consumers regarding the product's worth.

In the COVID-19 pandemic, MWDs are the best option for senior adults who need timely health monitoring at home. The MWDs manufacturing firms must develop and promote the MWDs with suitable advertising campaigns to highlight the benefits senior adults could gain from MWDs, promoting confidence in their health conditions. Moreover, higher acceptance of MWDs among senior adults reduces the burden on the healthcare system and helps maintain the availability of healthcare services to critical COVID-19 patients. Reducing the number of senior adults visiting the hospitals also decreases the likelihood of contracting COVID-19.

### Limitations

Despite the current research providing critical theoretical and practical contributions for researchers, users, and the healthcare industry, a few limitations were present in the study. As for the first limitation, due to the COVID-19 pandemic, the questionnaires were distributed online. As a result, the senior adults struggled to understand the questionnaire and provide prompt answers to the questions. It was also possible for the respondents to not be able to provide correct answers. Therefore, future research must incorporate face-to-face and online data collection modes to obtain complete responses from senior adults. Second, the data were only collected from senior adults in China and had limited generalization. Thus, it is suggested that future studies collect data from different geographic locations to improve the model generalization and enhance model predictive power. Future studies must add relevant factors of perceived value and mass adoption of MWDs to explore the adoption of MWDs. Finally, the current study explored the intention and adoption of MWDs among senior adult consumers while not evaluating the continuous intention of using MWDs. The exploration of continuous intention for MWDs enables the healthcare industry to enrich the MWDs and develop the continuous intention of using the MWDs, reduces the burden in the healthcare industry, and facilitates the senior adults.

## Data availability statement

The original contributions presented in the study are included in the article/Supplementary material, further inquiries can be directed to the corresponding author.

## Ethics statement

Ethical review and approval was not required for the study on human participants in accordance with the local legislation and institutional requirements. The patients/participants provided their written informed consent to participate in this study.

## Author contributions

ZX, MA, LS, and NH: conceptualization, methodology, data collection, writing—original draft. AM and QY: conceptualization, formal analysis, and writing revisions. All authors contributed to the article and approved the submitted version.

## Conflict of interest

The authors declare that the research was conducted in the absence of any commercial or financial relationships that could be construed as a potential conflict of interest.

## Publisher's note

All claims expressed in this article are solely those of the authors and do not necessarily represent those of their affiliated organizations, or those of the publisher, the editors and the reviewers. Any product that may be evaluated in this article, or claim that may be made by its manufacturer, is not guaranteed or endorsed by the publisher.
