# Supplementary material for: Modeling the adoption of medical wearable devices among the senior adults: Using hybrid SEM-neural network approach
Source: Front Public Health. 2022 Oct 28;10:1016065. doi: 10.3389/fpubh.2022.1016065 (PMC9650156; doi:10.3389/fpubh.2022.1016065)
Supplement: Supplementary file 1 [file Table_1.DOCX]

**Supplementary Files** - **Appendix 1.** Survey Questionnaire

| **Code** | **Question** | **Source** |
| --- | --- | --- |
| HIE1 | I think using wearable healthcare devices helped manage health. | Davis et al. (1989); Venkatesh et al. (2003); Cimperman et al. (2016); Talukder et al. (2020) |
| HIE2 | I think using wearable healthcare devices helped make life convenient. |  |
| HIE3 | I think using wearable healthcare devices helped me lead a regular lifestyle. |  |
| HIE4 | I think using wearable healthcare devices can be improve the quality of my daily healthcare activities. |  |
| EEX1 | I find wearable healthcare devices is easy to use. | Talukder et al. (2020);  Binyamin and Hoque (2020) |
| EEX2 | I think it would be easy for me to become skillful at using wearable healthcare devices. |  |
| EEX3 | I think that interaction with wearable healthcare devices is clear and understandable. |  |
| EEX4 | I think it would be quick for me to learn how to use wearable healthcare devices. |  |
| PRV1 | I think that wearable healthcare devices are reasonably priced. | Venkatesh et al. (2012) |
| PRV2 | I think that wearable healthcare devices are a good value for the money. |  |
| PRV3 | At the current price, I think wearable healthcare devices provides a good value. |  |
| PRV4 | Overall, I think wearable healthcare devices it is worth the price. |  |
| PVU1 | It is critical to check vitals and keep tabs on my health regularly. | Beh et al. (2019); Gao et al. (2015) |
| PVU2 | I may suffer some types of medical diseases. |  |
| PVU3 | I may suffer the chronic diseases. |  |
| PVU4 | I am at risk for suffering the stated problems above. |  |
| PVU5 | It is likely that I will suffer the stated problems above. |  |
| HCO1 | I think living life in the best possible health is very important to me. | Dutta-Bergman (2009) |
| HCO2 | I think my health depends on how well I take care of myself. |  |
| HCO3 | I actively try to prevent disease and illness by using wearable healthcare devices. |  |
| HCO4 | I think exercising, and taking preventive measures by using wearable healthcare devices is important for me to stay healthy. |  |
| PRE1 | I obtain accurate and error free services from wearable healthcare devices service providers. | Alam et al. (2020); Walker et al. (2002) |
| PRE2 | I think I can rely on the service provided by wearable healthcare devices service provider. |  |
| PRE3 | I think the service of wearable healthcare devices is consistent over the time. |  |
| PRE4 | I think services of wearable healthcare devices maintain standard continuously. |  |
| PRE5 | I think that wearable healthcare devices operate reliably and can satisfy me. |  |
| IWPD1 | I intend to use wearable healthcare devices to manage my health in the future. | Gao et al. (2015); Alam et al. (2020); Wang et al. (2020) |
| IWPD2 | I intend to use wearable healthcare devices to manage my health in my daily life in the future. |  |
| IWPD3 | I plan to use wearable healthcare devices frequently to manage my health in the future. |  |
| IWPD4 | I would be willing to develop a habit to use wearable health devices soon. |  |
| IWPD5 | I predict I would use wearable healthcare devices to manage my health information. |  |
| AWPD1 | I am actively using wearable healthcare devices to monitor my personal health. | Aksoy et al., (2020); Alam et al. (2020); Lee and Lee (2020) |
| AWPD2 | I use wearable healthcare devices technology to keep my health safe. |  |
| AWPD3 | I use wearable healthcare devices to manage my personal health on regular basis |  |
| AWPD4 | I trust in using wearable healthcare devices to manage my personal health. |  |

**Supplementary Files** - **Appendix 2.** Discriminant Validity

|  | **HIE** | **EEX** | **PRV** | **PVL** | **HCO** | **PRE** | **IMWD** | **AMWD** |
| --- | --- | --- | --- | --- | --- | --- | --- | --- |
| **Fornell-Larcker Criterion** |  |  |  |  |  |  |  |  |
| HIE | 0.722 |  |  |  |  |  |  |  |
| EEX | 0.686 | 0.728 |  |  |  |  |  |  |
| PRV | 0.665 | 0.716 | 0.736 |  |  |  |  |  |
| PVL | 0.710 | 0.711 | 0.712 | 0.724 |  |  |  |  |
| HCO | 0.703 | 0.663 | 0.670 | 0.671 | 0.711 |  |  |  |
| PRL | 0.666 | 0.682 | 0.675 | 0.659 | 0.651 | 0.719 |  |  |
| IMWD | 0.676 | 0.654 | 0.699 | 0.695 | 0.659 | 0.708 | 0.738 |  |
| AMWD | 0.682 | 0.707 | 0.704 | 0.710 | 0.660 | 0.685 | 0.692 | 0.720 |
| **Loading and Cross-Loading** |  |  |  |  |  |  |  |  |
| HIE1 | ***0.744*** | 0.497 | 0.497 | 0.537 | 0.526 | 0.483 | 0.519 | 0.477 |
| HIE2 | ***0.715*** | 0.478 | 0.480 | 0.553 | 0.474 | 0.503 | 0.486 | 0.532 |
| HIE3 | ***0.718*** | 0.493 | 0.459 | 0.524 | 0.514 | 0.528 | 0.525 | 0.494 |
| HIE4 | ***0.712*** | 0.513 | 0.485 | 0.513 | 0.505 | 0.499 | 0.492 | 0.483 |
| EEX1 | 0.467 | ***0.689*** | 0.498 | 0.547 | 0.480 | 0.492 | 0.453 | 0.471 |
| EEX2 | 0.498 | ***0.742*** | 0.549 | 0.494 | 0.486 | 0.562 | 0.522 | 0.506 |
| EEX3 | 0.500 | ***0.718*** | 0.508 | 0.523 | 0.449 | 0.489 | 0.493 | 0.501 |
| EEX4 | 0.530 | ***0.760*** | 0.528 | 0.560 | 0.478 | 0.549 | 0.524 | 0.546 |
| PRV1 | 0.485 | 0.547 | ***0.785*** | 0.561 | 0.526 | 0.561 | 0.565 | 0.539 |
| PRV2 | 0.491 | 0.494 | ***0.719*** | 0.556 | 0.463 | 0.551 | 0.526 | 0.478 |
| PRV3 | 0.468 | 0.543 | ***0.713*** | 0.515 | 0.457 | 0.510 | 0.494 | 0.515 |
| PRV4 | 0.515 | 0.527 | ***0.727*** | 0.525 | 0.517 | 0.504 | 0.539 | 0.501 |
| PVU1 | 0.537 | 0.519 | 0.531 | ***0.691*** | 0.431 | 0.537 | 0.541 | 0.524 |
| PVU2 | 0.535 | 0.504 | 0.526 | ***0.697*** | 0.521 | 0.536 | 0.528 | 0.505 |
| PVU3 | 0.503 | 0.511 | 0.496 | ***0.725*** | 0.472 | 0.493 | 0.509 | 0.502 |
| PVU4 | 0.474 | 0.523 | 0.507 | ***0.693*** | 0.480 | 0.494 | 0.482 | 0.476 |
| PVU5 | 0.548 | 0.519 | 0.530 | ***0.731*** | 0.538 | 0.575 | 0.539 | 0.488 |
| HCO1 | 0.466 | 0.421 | 0.488 | 0.417 | ***0.683*** | 0.409 | 0.404 | 0.428 |
| HCO2 | 0.493 | 0.412 | 0.437 | 0.490 | ***0.709*** | 0.481 | 0.472 | 0.449 |
| HCO3 | 0.538 | 0.520 | 0.505 | 0.587 | ***0.749*** | 0.514 | 0.508 | 0.526 |
| HCO4 | 0.489 | 0.488 | 0.472 | 0.457 | ***0.701*** | 0.474 | 0.482 | 0.469 |
| PRE1 | 0.498 | 0.451 | 0.473 | 0.534 | 0.444 | ***0.670*** | 0.468 | 0.507 |
| PRE2 | 0.468 | 0.511 | 0.523 | 0.451 | 0.468 | ***0.683*** | 0.534 | 0.480 |
| PRE3 | 0.484 | 0.487 | 0.468 | 0.527 | 0.453 | ***0.737*** | 0.561 | 0.535 |
| PRE4 | 0.470 | 0.514 | 0.477 | 0.481 | 0.454 | ***0.698*** | 0.516 | 0.497 |
| PRE5 | 0.613 | 0.645 | 0.669 | 0.705 | 0.588 | ***0.850*** | 0.694 | 0.678 |
| IMWD1 | 0.497 | 0.508 | 0.507 | 0.504 | 0.428 | 0.531 | ***0.689*** | 0.505 |
| IMWD2 | 0.518 | 0.485 | 0.534 | 0.527 | 0.506 | 0.537 | ***0.730*** | 0.542 |
| IMWD3 | 0.516 | 0.511 | 0.567 | 0.560 | 0.470 | 0.557 | ***0.733*** | 0.531 |
| IMWD4 | 0.448 | 0.451 | 0.464 | 0.498 | 0.430 | 0.559 | ***0.707*** | 0.509 |
| IMWD5 | 0.511 | 0.481 | 0.491 | 0.526 | 0.510 | 0.547 | ***0.696*** | 0.488 |
| AMWD1 | 0.484 | 0.485 | 0.573 | 0.548 | 0.481 | 0.546 | 0.517 | ***0.720*** |
| AMWD2 | 0.475 | 0.501 | 0.408 | 0.491 | 0.454 | 0.516 | 0.499 | ***0.733*** |
| AMWD3 | 0.506 | 0.530 | 0.531 | 0.494 | 0.484 | 0.530 | 0.532 | ***0.719*** |
| AMWD4 | 0.511 | 0.490 | 0.472 | 0.500 | 0.483 | 0.553 | 0.540 | ***0.710*** |

**Note:** HIE: Health Improvement Expectancy; EEX: Effort Expectancy; PRV: Price Value; PVU: Perceived Vulnerability; HCO: Health Consciousness; PRE: Perceived Reliability; IMWD: Intention to Use MWD; AMWD: Usage of MWD

**Source:** Author’s data analysis

**Supplementary Files** - **Appendix 3.** Average synaptic weights of the input and hidden neurons of the ANN

| **Network** |  | **Bias** | **HIE** | **EEX** | **PRV** | **PVU** | **HCO** | **PRE** |  | **Bias** | **IMWD** |
| --- | --- | --- | --- | --- | --- | --- | --- | --- | --- | --- | --- |
| *Factors effecting IMWD* | | *Hidden Layer* | | | | | |  | *Output Layer* | | |
| 1 | H(1:1) | -1.762 | -0.311 | 0.093 | -0.222 | -0.429 | 0.579 | -0.461 |  | -1.212 | -2.41 |
|  | H(1:2) | -1.207 | -0.151 | -0.112 | -0.108 | -0.158 | 0.386 | 0.161 |  |  | 1.055 |
| 2 | H(1:1) | -0.035 | -0.076 | -0.323 | -0.064 | 0.007 | 0.15 | 0.359 |  | -1.64 | 1.018 |
|  | H(1:2) | -0.545 | -0.637 | -0.494 | 0.192 | -0.799 | -0.04 | 0.09 |  |  | 0.375 |
|  | H(1:3) | 1.45 | 0.063 | 0.164 | 0.183 | 0.02 | 0.151 | 0.134 |  |  | 2.328 |
|  | H(1:4) | -0.176 | 0.368 | 0.442 | 0.001 | 0.258 | -0.338 | -0.401 |  |  | 0.764 |
| 3 | H(1:1) | 0.36 | -0.218 | 0.272 | 0.061 | 0.012 | 0.103 | -0.039 |  | -1.692 | 1.125 |
|  | H(1:2) | -1.458 | 0.027 | 0.02 | -0.272 | -0.199 | 0.239 | -0.455 |  |  | -1.342 |
|  | H(1:3) | 0.829 | -0.157 | 0.216 | 0.063 | 0.134 | 0.045 | 0.081 |  |  | 0.879 |
|  | H(1:4) | -0.464 | 0.427 | -0.471 | -0.12 | -0.044 | -0.05 | 0.118 |  |  | 1.115 |
|  | H(1:5) | 0.351 | 0.235 | 0.312 | 0.382 | -0.32 | -0.175 | -0.13 |  |  | 0.309 |
| 4 | H(1:1) | -0.132 | 0.171 | -0.065 | -0.373 | 0.264 | -0.493 | 0.287 |  | -1.651 | -1.597 |
|  | H(1:2) | -1.78 | -0.197 | -0.16 | -0.324 | -0.021 | 0.305 | -0.27 |  |  | -1.805 |
|  | H(1:3) | 0.119 | -0.238 | 0.099 | 0.386 | -0.378 | 0.417 | -0.338 |  |  | -1.555 |
| 5 | H(1:1) | -1.231 | 0.014 | -0.375 | -0.18 | -0.157 | 0.397 | -0.388 |  | -0.493 | -1.59 |
|  | H(1:2) | 0.203 | -0.188 | 0.177 | 0.293 | -0.108 | -0.219 | -0.006 |  |  | -1.061 |
|  | H(1:3) | -0.38 | 0.101 | 0.23 | 0.319 | -0.239 | -0.291 | 0.103 |  |  | 0.989 |
|  | H(1:4) | -0.307 | -0.38 | -0.227 | -0.035 | 0.275 | 0.452 | 0.138 |  |  | 0.726 |
|  | H(1:5) | -0.185 | 0.157 | 0.138 | 0.115 | 0.371 | 0.448 | 0.443 |  |  | -0.429 |
|  | H(1:6) | 0.263 | -0.373 | 0.209 | -0.359 | -0.251 | -0.379 | -0.435 |  |  | -0.389 |
| 6 | H(1:1) | -0.103 | -0.245 | 0.379 | -0.059 | -0.403 | 0.282 | 0.203 |  | -1.675 | 0.899 |
|  | H(1:2) | 0.123 | 0.424 | -0.087 | 0.248 | 0.186 | -0.685 | -0.034 |  |  | 1.117 |
|  | H(1:3) | -0.025 | -0.168 | -0.412 | -0.031 | 0.203 | 0.39 | 0.11 |  |  | 1.051 |
|  | H(1:4) | 1.7 | 0.003 | 0.463 | 0.029 | 0.006 | 0.309 | -0.1 |  |  | 1.855 |
|  | H(1:5) | 0.288 | 0.06 | 0.125 | -0.18 | 0.311 | -0.084 | -0.018 |  |  | 0.128 |
| 7 | H(1:1) | -1.606 | -0.199 | 0.034 | -0.252 | -0.054 | 0.218 | -0.506 |  | -1.935 | -2.282 |
| 8 | H(1:1) | -1.007 | 0.005 | -0.136 | -0.134 | -0.307 | 0.314 | 0.155 |  | -1.436 | 1.114 |
|  | H(1:2) | -1.692 | -0.14 | -0.075 | -0.356 | -0.289 | 0.472 | -0.373 |  |  | -2.633 |
| 9 | H(1:1) | 2.644 | 0.23 | -0.393 | 0.792 | 0.57 | -0.669 | 0.718 |  | -1.929 | 2.163 |
| 10 | H(1:1) | -1.952 | -0.098 | -0.218 | -0.234 | -0.063 | -0.241 | -0.117 |  | -2.139 | -2.329 |
| **Contribution** |  |  | **-0.747** | **0.055** | **-0.221** | **-0.501** | **1.105** | **-0.853** |  |  | **-3.542** |
|  |  |  | | | | | |  |  | | |
| **Network** |  | **Bias** | **IMWD** | **AMI** | **PEC** |  | **Bias** | **AMWD** |  |  |  |
| *Factors effecting AMWD* | | *Hidden Layer* | | | |  | *Output Layer* | |  |  |  |
| 1 | H(1:1) | 2.305 | 0.786 | 0.284 | 0.016 |  | -2.385 | 2.58 |  |  |  |
|  | H(1:2) | -0.006 | 0.236 | -0.087 | 0.129 |  |  | 0.154 |  |  |  |
| 2 | H(1:1) | -2.062 | -0.858 | 0.035 | 0.074 |  | -1.847 | -2.102 |  |  |  |
| 3 | H(1:1) | 2.399 | 0.911 | 0.166 | -0.205 |  | -2.105 | 2.326 |  |  |  |
| 4 | H(1:1) | -2.276 | -0.796 | -0.092 | 0.169 |  | -2.261 | -2.474 |  |  |  |
| 5 | H(1:1) | -0.195 | -0.036 | -0.02 | 0.031 |  | -2.109 | 1.567 |  |  |  |
|  | H(1:2) | -1.759 | -0.636 | -0.114 | 0.151 |  |  | -2.759 |  |  |  |
|  | H(1:3) | 0.282 | 0.362 | -0.123 | -0.368 |  |  | -0.177 |  |  |  |
| 6 | H(1:1) | 0.738 | -0.16 | -0.025 | -0.169 |  | -1.829 | -1.003 |  |  |  |
|  | H(1:2) | 1.778 | 0.601 | 0.113 | -0.286 |  |  | 2.847 |  |  |  |
|  | H(1:3) | -0.181 | 0.265 | 0.376 | 0.08 |  |  | -0.226 |  |  |  |
|  | H(1:4) | 0.074 | -0.455 | -0.217 | 0.488 |  |  | 0.016 |  |  |  |
| 7 | H(1:1) | -2.211 | -0.868 | -0.209 | 0.277 |  | -2.078 | -2.325 |  |  |  |
|  | H(1:2) | 0.022 | -0.154 | -0.069 | 0.125 |  |  | 0.077 |  |  |  |
|  | H(1:3) | 0.372 | -0.332 | -0.311 | 0.385 |  |  | 0.158 |  |  |  |
| 8 | H(1:1) | -2.43 | -0.801 | -0.049 | 0.135 |  | -2.478 | -2.644 |  |  |  |
| 9 | H(1:1) | 2.43 | 0.674 | 0.056 | -0.09 |  | -2.885 | 3.09 |  |  |  |
| 10 | H(1:1) | -2.422 | -0.8 | -0.096 | -0.025 |  | -2.37 | -2.545 |  |  |  |
| **Contribution** |  |  | **-2.304** | **-0.668** | **0.703** |  |  | **-5.491** |  |  |  |

**Note:** HIE: Health Improvement Expectancy; EEX: Effort Expectancy; PRV: Price Value; PVU: Perceived Vulnerability; HCO: Health Consciousness; PRE: Perceived Reliability; IMWD: Intention to Use MWD; AMWD: Usage of MWD; PEC: Pre-Existing Conditions; AMI: Average Monthly Income

**Source:** Author’s data analysis
